# The immune‐related, TGA1 redox‐switch: to be or not to be?

**DOI:** 10.1111/nph.16785

**Published:** 2020-07-29

**Authors:** Yuan Li, Gary J. Loake

**Affiliations:** ^1^ Institute of Molecular Plant Sciences School of Biological Sciences University of Edinburgh Edinburgh EH9 3BF UK; ^2^ Synthetic and Systems Biology School of Biological Sciences University of Edinburgh The King's Buildings Edinburgh EH9 3BF UK

**Keywords:** cellular signalling, cysteine (Cys) residues, NON‐EXPRESSOR OF PR GENES (NPR1), pathogen, plant immune responses, redox switch, salicylic acid (SA), TGA transcription factors

A key feature following attempted pathogen ingress is the marked and rapid production of reactive oxygen and nitrogen intermediates (ROI and RNI, respectively), the so‐called oxidative and nitrosative burst (Grant & Loake, 2000; Besson‐Bard *et al*., 2008). These molecules are directly antimicrobial, drive the oxidative cross‐linking of cell wall structural proteins, cue the development of programmed cell death in directly challenged cells and orchestrate a plethora of immune responses surrounding the site of attempted pathogen ingress (Torres *et al*., 2002; Yu *et al*., 2014).

‘While this regulatory circuitry is currently rather under‐appreciated within the plant–microbe field, the future manipulation of these redox‐switches will provide unique opportunities to reconfigure plant immune responses.’

In the context of cellular signalling, redox‐switches embedded within target proteins enable pathogen‐triggered ROI/RNI to regulate protein function, for example, by modulating enzyme activity, DNA/RNA binding, ion translocation, protein localization and especially pertinent here, protein–protein interactions (Spadaro *et al*., 2010; Chi *et al*., 2013). While this regulatory circuitry is currently rather under‐appreciated within the plant–microbe field, the future manipulation of these redox‐switches will provide unique opportunities to reconfigure plant immune responses.

The plant immune activator, salicylic acid (SA), which accumulates in response to attempted pathogen ingress, drives the expression of an extensive suite of immune‐related genes (Durrant & Dong, 2004). Key players in the induction of these SA‐dependent genes are the transcriptional co‐activator, NON‐EXPRESSOR OF PR GENES (NPR1) (Fu *et al*., 2012; Ding *et al*., 2018) and the basic domain leucine zipper (bZip) transcription factors TGACG‐BINDING FACTOR 1/4 (TGA1/TGA4) (Zhang *et al*., 2003), together with closely related paralogs of both of these molecular components. Nuclear localized NPR1 is thought to physically interact with TGA1/TGA4 to promote binding at cognate *cis*‐elements located within the promoters of target genes, in an SA‐dependent fashion (Zhang *et al*., 1999).

Previous findings have suggested the presence of an intramolecular disulphide (S–S) bond between cysteines (Cys) 260 and Cys266 and Cys172 and Cys287 of TGA1 (Després *et al*., 2003; Lindermayr *et al*., 2010). SA has been proposed to induce reduction and associated breakage of these disulphide bonds, reconstituting thiol (–SH) formation at each relevant Cys residue (Fig. 1), enabling TGA1 to interact with NPR1. This embedded redox‐switch therefore regulates the interaction of TGA1 with NPR1 and by extension the expression of SA‐, NPR1‐, and TGA1/4‐dependent genes, presumably through modulating a change in the structural confirmation of TGA1. The existing evidence therefore placed a redox‐switch at the heart of TGA1 function.

**Fig. 1:**
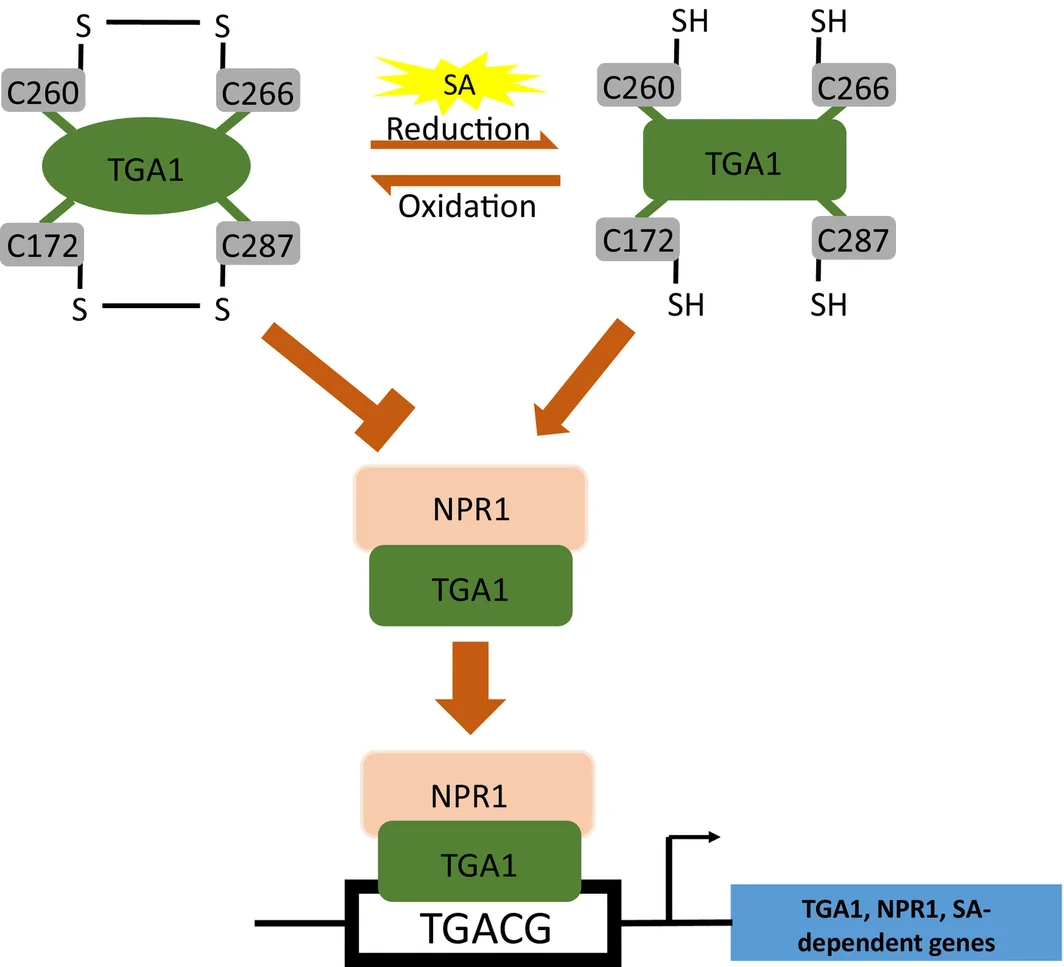
The TGACG‐BINDING FACTOR 1 (TGA1) redox‐switch controls the expression of TGA1‐, NON‐EXPRESSOR OF PR GENES (NPR1)‐, and salicylic acid (SA)‐dependent genes. When oxidized, TGA1 forms intramolecular disulphide bridges between cysteine (Cys) 260 and Cys266 and also between Cys172 and Cys287. Following reduction, the two TGA1 intramolecular disulphide bridges are broken, resulting in formation of thiols at the corresponding Cys residues, leading to a conformational change. The resulting change in TGA1 structure facilitates interaction with NPR1, enabling the binding of TGA1 to its cognate *cis*‐element, supporting transcriptional activation of TGA1‐, NPR1‐, and SA‐dependent genes.

In contrast to previous reports, Budimir *et al*. (2020; doi: 10.1111/nph.16614), in an article published in *New Phytologist,* now claim redox active Cys residues do not function in SA‐ or pathogen‐induced expression of TGA1‐regulated genes. To provide a platform to explore whether the redox‐modulated, NPR1‐dependent, DNA‐binding activity of TGA1 influences the expression of SA‐dependent target genes, Budimir *et al*. first elegantly identified a suite of SA‐induced TGA1/TGA4‐dependent genes employing RNAseq‐based approaches. This careful analysis identified that 193 out of 2090 SA‐inducible genes required TGA1/TGA4 function for maximal expression post‐exogenous SA application. As an interesting aside, one of the most robustly uncovered SA‐induced, TGA1/TGA4‐dependent genes encoded the SA hydroxylase DOWNY MILDEW RESISTANT 6‐LIKE OXYGENASE 1 (DLO1), linking TGA1/TGA4 activity to SA catabolism (Budimir *et al*., 2020). The identified gene network now provided an effective read‐out of the breadth of TGA1/TGA4 function. The relatively low frequency of SA‐inducible genes under the control of TGA1/TGA4 was somewhat smaller than expected and maybe explained by data showing the expression pattern of TGA1/TGA4, which indicated that the cognate promoters are predominantly active only in vascular tissue (Wang *et al*., 2019).

With the identification of a suite of SA‐induced, TGA1/TGA4‐dependent genes, Budimir *et al*. were now in a position to express a TGA1 variant that possessed amino acid substitutions in all four Cys residues (C172N, C260N, C266S, C287S), to assess the impact of an absence of redox regulation on this key defence‐related transcriptional activator. The authors were surprised to discover that this TGA1 quadruple Cys mutant partially complemented an *Arabidopsis tga1 tga4* double mutant line with respect to exogenous SA application, scored by monitoring the expression of the selected marker genes *DLO1*, *Glutathione S‐transferase F6* (GS*TF6*) and *BETA‐1,3‐GLUCANASE* (*BGL2*). Significantly, the activity of the expressed TGA1 quadruple Cys mutant was as effective as the expressed wild‐type TGA1 control. The inability of either gene to convey complete complementation was ascribed to the absence of TGA4, as only TGA1 and not both TGA1 and TGA4 were expressed in the *tga1 tga4* double mutant. Alternatively, the presence of an N‐terminal HA‐tag in the expressed transgenes may have limited TGA1 function. Importantly, Budimir *et al*. therefore concluded that the SA‐dependent redox‐switch embedded within TGA1 was not required for the TGA1/SA/NPR1‐dependent expression of TGA1/TGA4 target genes.

In a similar fashion, Budimir *et al*. determined the expression of *SARD1* (Zhang *et al*., 2010), an SA/NPR1/TGA1/TGA4‐dependent target gene, in the *tga1 tga4* double mutant challenged with the virulent bacterium, *Pseudomonas maculicola* (*Psm*), following expression of the TGA1 quadruple Cys mutant. Again, expression of the employed marker gene, *SARD1*, was induced to a similar extent by both the *TGA1* quadruple Cys mutant and a wild‐type *TGA1* transgene. Thus, again Budimir *et al*. posit that the absence of the SA‐dependent redox‐switch embedded within TGA1 does not impact the regulatory properties of this transcriptional activator.

These observations sharply contrast with previous findings (Després *et al*., 2003; Lindermayr *et al*., 2010), which as highlighted earlier, uncovered an SA‐dependent redox‐switch integral to TGA1 function. Why is there such an apparent discrepancy between these claims? First, it is important to emphasize that the TGA1 SA‐dependent redox‐switch controls formation of an intra‐molecular disulphide (S–S) bond between Cys260 and Cys266 and probably also Cys172 and Cys187 (Després *et al*., 2003; Lindermayr *et al*., 2010). The establishment of these disulphides is thought to sterically preclude the interaction of TGA1 with NPR1 and, by extension, the optimal transcriptional activation function of TGA1. The reduction of these disulphides to the respective thiols (–SH) breaks these Cys‐dependent intra‐molecular linkages, altering the structure of TGA1, enabling interaction with NPR1 (Després *et al*., 2003). The TGA1 quadruple Cys mutant is therefore presumably already in a conformation compatible with NPR1 interaction, analogous to reduced wild‐type TGA1, where the two disulphide linkages have been broken. Hence, the TGA1 quadruple Cys mutant likely mimics the reduced and active form of TGA1, rather than blocking the redox‐switch that generates active TGA1.

It may therefore not be so surprising that this TGA1 quadruple mutant drives expression of the scored marker genes. Perhaps, a more pertinent question to ask is: does this TGA1 mutant induce the selected marker genes to a *greater* extent than the wild‐type protein, as 100% of the TGA1 Cys quadruple mutant will presumably occupy the active conformation? Interestingly, the data from Budimir *et al*. suggest this is not the case: the magnitude of marker gene expression established by the wild‐type and mutant transgenes is similar. This data is actually comparable to the previous findings that show a TGA1 quadruple Cys mutant expressed from a *35S* promoter did not increase expression of the SA‐dependent genes *PR1*, *PR2* and *PR5* in a *tga1 tag4* double mutant (Lindermayr *et al*., 2010). However, a TGA C172S and C287S double mutant increased the expression of these *PR* genes by less than two‐fold (Lindermayr *et al*., 2010), a borderline level of significance. The failure of these reduced Cys mimic mutants to strongly enhance SA‐dependent gene expression may reflect the presence of robust negative regulatory mechanisms, a key feature of plant immunity (Frye *et al*., 2001; Yun *et al*., 2011). Alternatively or in addition, only a small percentage of the wild‐type TGA1 pool might be required to occupy the active conformation to drive maximal TGA1‐, SA‐, and NPR1‐dependent gene expression.

In their analysis of the TGA1 redox‐switch, Budimir *et al*. have also to date only monitored the expression of a small number of selected reporter genes. It would be interesting to utilize an RNAseq approach to monitor all TGA1/TGA4‐dependent genes to interrogate the full breadth of TGA1 function. Further, only one selected time point was selected for their analysis. Thus, it might be more informative if a time course analysis is undertaken. In addition, mutation of the four TGA1 Cys residues individually and in combination might be revealing, because the quadruple Cys mutant may disrupt, for example, TGA1 stability.

Perhaps the most powerful approach would be to utilize a TGA1 protein variant locked into the oxidized, inactive form, by generating fixed, disulphide‐like co‐valent bonds at the appropriate Cys residues (Xiang *et al*., 2013). If the potential redox‐switch embedded in TGA1 was indeed dispensable, this mutant TGA1 protein would still be expected to drive TGA1‐, SA‐, and NPR1‐dependent expression of target genes.

As Hamlet, Prince of Denmark famously said in Shakespeare's eponymous play: ‘to be or not to be, that is the question’. Likewise, it may be prudent to generate and interpret additional data before we can pen the obituary of the TGA1 redox‐switch.
